# Dental Inventions and Pretensions

**Published:** 1852-07

**Authors:** 


### ARTICLE V.

### *Dental Inventions and Pretensions.*

There is so much of unfounded assumption, and so much paltry jealousy abroad, that we cannot render more efficient service to the dental profession than to bring under its notice whatever may be calculated to compromise its character and lower its dignity, and we feel satisfied that the public odium, and public exposure, which inevitably follow these discreditable practices are the means of preventing these repetitions. The moment a valuable discovery is announced, there are a swarm of pretenders, or jump-up-behind persons, sure to start up and claim the merit and the honor and the profits of the invention, and the humble or modest man of genius, who may not be possessed of the necessary influence, or standing, to assert his rights, too often finds himself tricked out of them by unscrupulous swaggerers, and ignorant boasters; the last case of this sort that has come before the dental and medical profession in Europe is, the mechanical invention for the relief of congenital cleft palate, the value and importance of which will probably account for the avidity with which it has been laid hold of by several conflicting claimants. A brief statement of the facts connected with this matter will enable the profession to pronounce at once upon the claims of the several candidates and draw their own inferences.

It appears that in December, 1844, Professor Fergusson, of King's College, London, read a paper before the Medical Chirurgical Society, respecting his views upon "congenital cleft palate" and the operation of staphyloraphy. Having explained the nature of his *modus operandi*, he referred to the various mechanical apparatuses that had been employed, and exhibited a whole series of casts, with different kinds of palates for correcting these deformities, which had been invented and constructed by the late Mr. Alexander Nasmyth, who it is well known in England, had devoted great attention to the subject of mechanical contrivances for this purpose. During the period of experiments and ultimate improvements, he had collected a large number of casts of the mouths on which he had operated, and experimented with the mechanical apparatus and notes attached to each case. Four or five years afterwards, in consequence of ill health, Mr. Nasmyth disposed of his practice to a Mr. Edwin Saunders, who, as a matter of course, purchased, also, the stock in trade, models, drawings, notes of cases, the various mechanical contrivances which Mr. Nasmyth had invented or improved upon and left behind him. Since that period the world and the profession have heard nothing of the inventive genius of the *successors to his practice and his position*, and it is only when a new claimant comes forward in the shape of a pamphlet, by Mr. Mapleton, "on the treatment of cleft palate by mechanical means," that the profession becomes suddenly apprised of who is "the real Simon Pure," whether arising from innate modesty, or because it would be more convenient hereafter to repudiate assertions that cannot be supported, this said Mr. Edwin Saunders leaves the task of eulogy and remonstrance to his brother, a Mr. Josiah Saunders, who intimates that brother Edwin is *about* to publish *his* views upon the same subject, that he has no wish to keep back anything from the profession, (seeing that they are already informed upon the subject from two or three different sources,) but is merely influenced by a desire not to commit an immature work to the press.

Now, it does seem marvellously strange, that Mr. J. Saunders and his brother should have forgotten a very important fact connected with this controversy. When Mr. Edwin Saunders, through his *agent* Mr. Josiah Saunders, lays claim to this invention, he appears to have altogether overlooked the rights of his predecessor, Mr. Nasmyth, whose business he purchased, and whose casts, improvements, &c., fell into his hands—he appears further to have forgotten that so long ago as four or five years since, a Mr. Stearn, surgeon, waited upon him and submitted his invention, which was similar to the one now published—that Mr. Edwin Saunders who had then just succeeded to Mr. Nasmyth's practice, on being applied to by a brother practitioner respecting the merits of Mr. Stearn's invention, was told in reply, that "it did not answer." No sooner, however, does a new claimant appear in the field and assert his right and the publication of a pamphlet, than Mr. Saunders comes forward and assures the profession that he is "the real Simon Pure," as he is appropriately termed in the *London Medical Times*. We believe, however, that neither Mr. Saunders nor Mr. Mapleton have the slightest claim to the honor of the invention, and we shall establish this position, we believe, most conclusively, by an examination of the peculiarities of each, and then leave the profession to judge of the candor and integrity of Mr. Edwin Saunders. Dr. Harris, in his work, page 781, describes Stearn's instrument as follows: "Consisting, first, of a gold plate fitted to the vault of the palate in the usual manner, and to the upper and posterior margin of which, is attached a flat spiral spring, admitting of easy vibration, backwards and forwards, to the posterior extremity of which is attached a flexible velum of *caoutchouc*." Now, Mr. Mapleson's plan, as described in his pamphlet, page 12, is as follows: "A gold plate made to fit the roof as far back as the mouth would permit, to the pharyngeal part of this instrument was suspended a movable uvula composed of *gold*, and acted upon by means of a spring attached to the posterior surface of the gold plate." There is a very ingenius variation of phraseology in the description of the recent *invention*, which, with the slight difference in the use of the material employed, means precisely the invention of Mr. Stearn, and nothing more nor less, notwithstanding the great attention that Mr. Josiah Saunders assures us his brother has paid to this subject, it will strike every one as somewhat extraordinary, that he makes no reference whatever to Mr. Stearn's invention—that he makes no allusion to the improvements which Mr. Nasmyth, during his professional career introduced and perfected. His great genius appears to have had stomach for them all—he swallows everything into his capacious maw, and after digesting the views, the opinions, the mechanical contrivances and improvements of all who have preceded him, he comes forward, at the eleventh hour, disgorges the mass, and asks the profession to believe that it is his own. This, however, is presuming too much upon the credulity and common sense of the profession and the public. We all know that this said Mr. Edwin Saunders is not exactly the person to let his light remain hidden, either under a bushel, or his own mahogany, even it were only a farthing rush-light, and we must therefore infer that he had some very stringent reasons of his own for keeping this discovery secret. It is just possible that his delicacy of feeling may have arisen from the conviction that the time had not yet arrived when he could safely bring it forward as his own. Before the honors of the invention, thus barefacedly claimed by Mr. Saunders are awarded to him, it would be well if the profession were informed of the extent of the late Mr. Nasmyth's improvements, and it would be desirable, also, to ascertain in what respect Mr. Saunder's invention differs from that brought forward several years since by Mr. Stearn, which is still in existence, and the merits of which have been tested and examined by several dental practiciens. R.

### ARTICLE VI.

### *Dental Exhibition at the World's Fair.*

